# Fluoride pollution of atmospheric precipitation and its relationship with air circulation and weather patterns (Wielkopolski National Park, Poland)

**DOI:** 10.1007/s10661-012-2962-9

**Published:** 2012-11-01

**Authors:** Barbara Walna, Iwona Kurzyca, Ewa Bednorz, Leszek Kolendowicz

**Affiliations:** 1 Jeziory Ecological Station of Adam Mickiewicz University, P.O. Box 40, 62-050 Mosina, Poland; 2 Department of Water and Soil Analysis, Faculty of Chemistry, Adam Mickiewicz University, Drzymaly 24 Str., 60-613 Poznań, Poland; 3 Department of Climatology, Institute of Physical Geography and Environmental Planning, Adam Mickiewicz University, Dziegielowa 27 Str., 60-680 Poznań, Poland

**Keywords:** Fluoride, Wielkopolski National Park, Atmospheric circulation, Back trajectories

## Abstract

A 2-year study (2010–2011) of fluorides in atmospheric precipitation in the open area and in throughfall in Wielkopolski National Park (west-central Poland) showed their high concentrations, reaching a maximum value of 2 mg/l under the tree crowns. These high values indicate substantial deposition of up to 52 mg/m^2^/year. In 2011, over 51 % of open area precipitation was characterized by fluoride concentration higher than 0.10 mg/l, and in throughfall such concentrations were found in more than 86 % of events. In 2010, a strong connection was evident between fluoride and acid-forming ions, and in 2011, a correlation between phosphate and nitrite ions was seen. Analysis of available data on F^−^ concentrations in the air did not show an unequivocal effect on F^−^ concentrations in precipitation. To find reasons for and source areas of high fluoride pollution, the cases of extreme fluoride concentration in rainwater were related to atmospheric circulation and weather patterns. Weather conditions on days of extreme pollution were determined by movement of weather fronts over western Poland, or by small cyclonic centers with meteorological fronts. Macroscale air advection over the sampling site originated in the western quadrant (NW, W, and SW), particularly in the middle layers of the troposphere (2,500–5,000 m a.s.l.). Such directions indicate western Poland and Germany as possible sources of the pollution. At the same time in the lower troposphere, air inflow was frequently from the north, showing short distance transport from local emitters, and from the agglomeration of Poznań.

## Introduction

Environmental pollution with fluoride compounds is currently one of the most important problems because of its hazardous effect on ecosystems (ATSDR 2003; Divan et al. 2008; Weinstein and Davison 2004; WHO Report 2002). Fluoride enters the atmosphere mainly from anthropogenic sources. Natural sources like volcanic eruptions, rock dust, or the marine environment make only a small contribution to global atmospheric emission of this compound (Barnard and Nordstrom 1982; Friend 1989; Saether et al. 1995b). The principal anthropogenic sources include aluminum smelters, fertilizer factories, and industrial activities such as brick, tile, pottery and cement works, ceramic industries, and glass manufacture (Cape et al. 2003). Owing to government regulation, most fluoride emitters have recently been equipped with effective filters. However, emission of these compounds to the atmosphere continues to be a problem (Franzaring et al. 2006).

Anthropogenic fluorine emitted into the atmosphere is highly reactive and readily hydrolyzes to form hydrogen fluoride. This reacts with many materials (in both vapor phase and in aerosols), forming typically nonvolatile, stable fluorides (ATSDR 2003). Alternatively, anthropogenic fluoride emissions include hydrogen fluoride and particulate fluorides (Kirk and Lester 1986).

The distribution and deposition of airborne fluoride depend on various factors, such as level of emission (Yanchenko and Baranov 2010), particulate grain size (Sloof et al. 1989), and chemical reactivity of the species (Hara et al. 1998). They also depend on meteorological conditions such as temperature, wind direction, wind speed, turbulence, and precipitation (Gasic et al. 2010; Scheringer 2009). F^−^ compounds are removed from the atmosphere via dry and wet deposition. Atmospheric circulation is important in transporting fluorides over large distances, especially for stable and non-volatile fluorides adsorbed on particulate matter, as well as for smaller particles (diameter, <10 μm) of fluorides in aerosols (Sloof et al. 1989). Long-duration resistance of F^−^ compounds in the atmosphere can extend its transport distance. However, wet deposition of these compounds with atmospheric precipitation (rain and snow) and fog or other condensation products, resulting in cloud washout and atmospheric scavenging, is also of great importance for removal of these compounds from the atmosphere (Chate et al. 2003; Sloof et al. 1989).

Data concerning fluoride concentration in atmospheric precipitation over the last decade in Europe are limited. Moreover, air-monitoring institutions in Europe (government or international) do not investigate this parameter (e.g., EMEP/CCC Reports). In regions of human impact in Poland, the range of fluoride in precipitation was up to 0.56 mg/l during 2002–2004 (Walna and Kurzyca 2007). In 2004, Franzaring et al. (2006) measured fluoride concentration from <0.1 to 2.4 mg/l in bulk precipitation, near a chemical plant producing fluoride in Germany. He also observed higher fluoride concentrations near the emitter, mainly from the leeward side. Similar observations have been presented by Koblar et al. (2011).

Fluoride has important biological effects, especially on vegetation. This is because its compounds damage plants at concentrations about 1,000 times lower than those causing detectable human health effects (Manins et al. 2001). The lowest HF concentrations in air that produce visible injury are around 0.3 μg/m^3^, if exposure time is sufficiently long (Cape et al. 2003). Fluoride that has penetrated into a plant tissue affects its metabolism in a number of ways (e.g., Feng et al. 2003). Relationships between atmospheric fluoride and fluoride accumulation in plant leaves, and between fluoride in soil solution and fluoride taken up by plants, have been widely described (e.g., Doley 2010; Fangmeier et al. 2002; Karolewski et al. 2000; Klumpp et al. 1996; Koblar et al. 2011; Weinstein and Davison 2004). Permanent stress caused by industrial sources also has wider ecological consequences. It alters the structure and composition of plant communities, and causes fluoride to enter other links of the trophic network (McCune and Weinstein 2002). In areas subject to fluoride emissions, adverse effects have been observed in the bone systems of both animals and humans (ATSDR 2003; Weinstein and Davison 2004; WHO Report 2002). Toxic effects in the aquatic environment have also been reported (Camargo 2003; Zhang et al. 2007). One should also note the behavior of F^−^ in soil (Horner and Bell 1995; Koblar et al. 2011; Walna 2007). F^−^ forms many complex ions, e.g., AlF_x_^(3-x)^ (Frankowski and Ziola-Frankowska 2010), that migrate, and a substantial proportion of these enter groundwater (Saether et al. 1995a). Soils in regions affected by fluoride emissions show accumulations of this element (Arnesen et al. 1995; Horntvedt 1995). In soils contaminated with fluoride, the content of organic matter declines, as does the activity of microorganisms (Van Wensem and Adena 1991).

The aim of this research is to present results of fluoride determination in precipitation, in an open area and under tree crowns. Furthermore, we relate cases of extreme fluoride pollution of precipitation measured in the protected area of Wielkopolski National Park to atmospheric circulation and weather patterns.

## Site description

The research was carried out at Jeziory Ecological Station of Adam Mickiewicz University, within Wielkopolski National Park of west-central Poland (N 52°15′56″, E 16°48′06″). The metropolitan area of Poznań (about 600,000 inhabitants) is nearby (25 km), whose influences on the investigated area have been presented previously (Walna et al. 2004). There are a host of large and small industrial plants in the city, some of which emit fluorine compounds. Additionally, between the city and a rainwater collection site in the park, at a distance of about 12 km from the latter site, is the large Luvena chemical plant. This has been manufacturing phosphate fertilizers, hydrofluoric acid, and sulphuric acid for years. The plant’s many pro-ecological measures over the last 15 years have limited its environmental impact considerably. However, its cyclic production undoubtedly remains a source of air pollution. Its fluorine emission to the atmosphere decreased from 19,500 kg/year (1988), through 4,000 kg/year (2000), to about 2,000 kg/year (2010) (Ecological Report 2010/2011). Some studies of F compounds in soils from the vicinity of an aluminum smelter plant were described by Frankowski et al. (2010b). Studies of precipitation in the national park area are a continuation of previous research (Walna et al. 2004; Walna and Kurzyca 2007, 2009).

## Methodology

In the study period from January 2010 to December 2011, 134 precipitation samples were collected daily, in open terrain (forest clearing) and under the tree crown (oaks), in accordance with the ICP Forest Manual (2006). The following parameters were measured for each sample: precipitation amount (manually—Hellmann rain gauge), pH (Elmetron CP-315 pH meter), and electrical conductivity (Elmetron CC-311 conductivity meter). Concentrations of fluoride and the ions Cl^−^, NO_2_^−^, NO_3_^−^, PO_4_^3−^, SO_4_^2−^ and Na^+^, NH_4_^+^, K^+^, Mg^2+^, Ca^2+^ were also determined (Dionex ICS 1100 ion chromatograph; anion analysis: AS14 analytical column (4 × 250 mm), AG14 guard column (4 × 50 mm), ASRS-II 4-mm suppressor, and 1.8 mM Na_2_CO_3_/1.7 mM NaHCO_3_ with the flow rate 1.2 ml/min as an eluent; cation analysis: CS12A analytical column (4 × 250mm), CG12A guard column (4 × 50), CSRS-ULTRA 4-mm suppressor, and 20 mM mM methylenesulphonic acid with the flow rate 1.0 ml/min as an eluent). Method detection limits depended on the compounds being determined, and they varied in the range between 0.004 and 0.01 mg/l. The quantification limit for fluoride determination was 0.01 mg F^−^/l. Recoveries of known additions in samples were within 94–105 %. A good linear relationship between the peak area and the analyte concentration was obtained in a wide range of concentrations. During both sample collection and analysis, quality control and quality assurance procedures were applied. However, one should also note the analytical problems during fluoride determination using ion chromatography with isocratic elution. For samples (occurring mainly in spring) with elevated organic acids’ (formic, acetic, oxalic) concentrations, determination of fluoride can be impossible or uncertain (nearing retention time). For those cases, gradient elution must be applied (e.g., Zygmunt et al. 2012), or these data must be removed from the database (availed in this study). Daily fluoride concentrations in the air, from two sites at 2 km distance from the emitter, were extracted from the Luvena chemical plant report (Report: Air quality in Luboń 2011).

The relationship between fluoride concentration in atmospheric precipitation and the synoptic situation was analyzed for the precipitation samples collected in the open area, where it was possible to eliminate rinsing of the deposited pollutants by precipitation water. We analyzed the influence of trajectories of air advection on the general level of precipitation water pollution. For this purpose, the catalog of types of atmospheric circulation in western Poland of Niedźwiedź (2011) was used. Next, cases of extreme fluoride concentrations were distinguished. The 95th percentile served as a differentiating criterion. This method is in keeping inter alia with the approach recommended by the Intergovernmental Panel on Climate Change (IPCC 2001) for determining extreme weather events. A similar criterion was used in the Atlas of extreme meteorological phenomena and synoptic situations in Poland (Ustrnul and Czekierda 2009). For such distinguished days with extreme fluoride pollution, we analyzed the synoptic situation on the days preceding the occurrence of maximum concentration and on the day itself. To that end, we used synoptic maps of 00:00 UTC published by the Institute for Meteorology and Water Management in the Daily Meteorological Bulletins (2010, 2011). Maps of sea-level atmospheric pressure anomalies were also drawn. These maps show the difference between the average pressure distribution over Europe and the North Atlantic and its distribution on the day in question (Kalnay et al. 1996). The sea-level pressure data are from National Centers for Environmental Prediction/National Center for Atmospheric Research Reanalyses Project (NCEP/NCAR), available from 2010. The maps of pressure anomalies were accomplished with information from synoptic maps. Further, for each of the extreme fluoride pollution days, trajectories of air mass motion over the sampling point were analyzed over the previous 24 h, using the Hybrid Single Particle Lagrangian Integrated Trajectory Trajectory Model (Draxler and Rolph 2012; Rolph 2012). This model considers three heights above sea level, for which the trajectory analysis was done: 500 m (corresponding to the central mixing layer), 2,500 m (corresponding to the average height of isobaric surface 850 hPa), and 5,000 m (corresponding to the altitude of Rossby waves at mid-latitudes, affecting the spatial distribution of lower atmospheric circulation forms (Fortak 1971). The analysis of air motion trajectories at three altitudes was a significant confirmation and supplementation of the data obtained from synoptic maps, and permitted identification of probable fluoride emission areas.

## Results and discussion

**Fig. 1:**
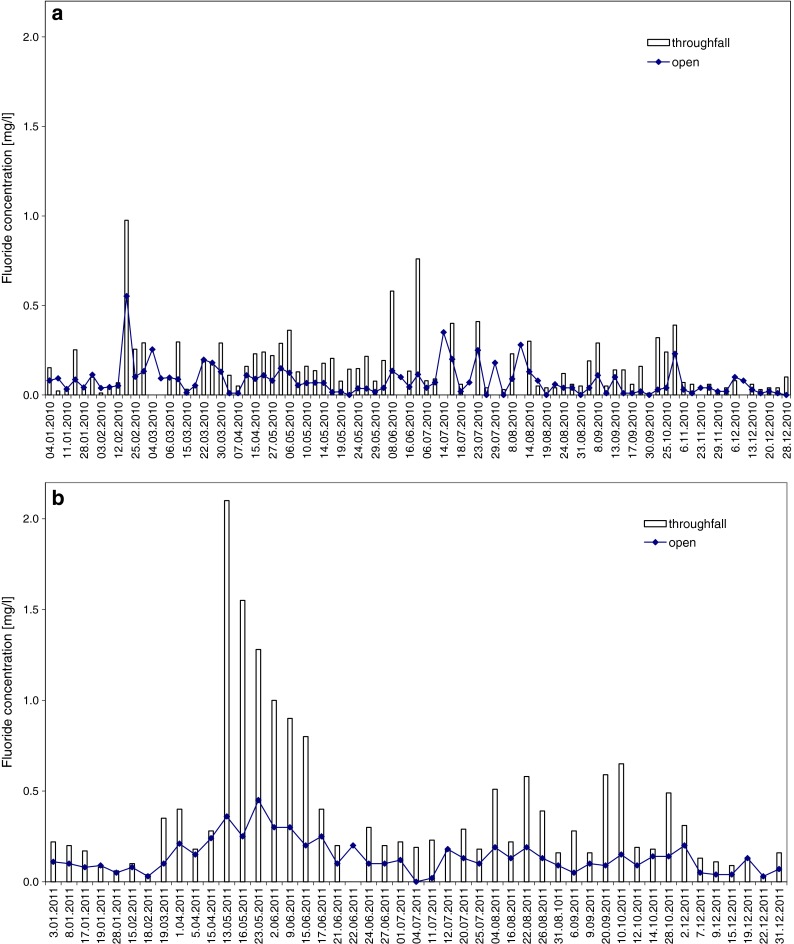
Fluoride concentrations in atmospheric precipitation in the open area and throughfall in 2010 (**a**) and 2011(**b**), Jeziory Ecological Station

Figure 1a, b shows annual patterns of fluoride concentrations in open terrain and under trees, for the years 2010 and 2011. The analyzed years differ markedly. In 2010, annual rainfall was much higher (755 mm) than the multi-year average (550 mm); annual fluoride concentrations in the open area and in throughfall were half those in 2011, for which total precipitation was less (436 mm; Table 1). Maximum concentration in throughfall in 2011 was twice as high as in 2010. In all these cases, rainwater collected under trees was richer in fluorides than in respective open-area samples. We observed on many occasions that minimum fluoride levels in open-area samples corresponded to high concentrations in throughfall (e.g., in October 2011 and June 2010). This indicates that dry deposition has a decisive share of total fluoride deposition. In 2011, over 51 % of precipitation in the open area had fluoride concentrations higher than 0.10 mg/l, and in throughfall, such concentrations were found in more than 86 % of events. Low fluoride concentrations (<0.04 mg/l) in 2010 were observed in 46 % of open-area samples, but only 13 % in 2011. Despite the significant rainfall difference between 2010 and 2011, such a distribution of concentrations resulted in very similar and extremely high fluoride deposition values for throughfall: 51 mg/m^2^/year. Deposition in the open area amounted to 30.4 mg/m^2^ in 2010, and 45.9 mg/m^2^ in 2011.

**Table 1:** Annual characteristics and correlation coefficients (*p* < 0.05) for rainfall in the open area and in the throughfall in 2010 and 2011 in the Wielkopolski National Park (Jeziory Ecological Station)

Annual characteristics	2010		2011	
	Open	Throughfall	Open	Throughfall
Average conc. (mg/l)	0.09 ± 0.01	0.16 ± 0.02	0.14 ± 0.01	0.34 ± 0.03
Weighted average (mg/l)	0.04 ± 0.01	0.10 ± 0.01	0.10 ± 0.01	0.23 ± 0.02
Concentration range (mg/l)	<0.01–0.55	0.01–0.98	<0.01–0.45	0.03–2.10
Number of samples	87	79	48	47
Rainfall (mm)	755	504	436	227
Deposition (mg/m^2^/year)	30.4	51.2	45.9	51.5
Correlation coeff. SO_4_^2−^/F^-^	**0.58**	**0.74**	0.30	0.18
Correlation coeff. NO_3_^−^/F^-^	**0.66**	**0.77**	**0.65**	0.22
Correlation coeff. Cl^−^/F^−^	**0.51**	**0.71**	0.30	0.24
Correlation coeff. NO_2_^−^/F^−^	0.42	0.47	**0.61**	**0.86**
Correlation coeff. PO_4_^3−^/F^−^	0.16	0.50	0.22	**0.55**

Observations of precipitation chemistry have been made by the Jeziory Ecological Station for many years (Walna et al. 2004; Walna and Kurzyca 2007, 2009). The characteristic feature of precipitation, apart from its location in the area of Wielkopolski National Park, is low average annual pH and large fluctuations of acid-forming ion concentrations. In 2010 and 2011, average precipitation pH was 4.51 and 4.72, respectively.

When analyzing non-fluoride contaminants in precipitation, one observes simultaneous maxima and similar annual patterns of sulphates, chlorides, and nitrates. This was also characteristic of the annual variability of these contaminants in previous years (Walna et al. 2004). In 2011, exceptionally high concentrations of these ions in throughfall were observed on 10 October and 2 December, respectively (milligrams per liter): 24 and 77 (sulphate), 42 and 25 (nitrate), and 18 and 24 (chloride). Low pH accompanied by a high content of acid-forming ions has an interactive effect on vegetation and soil. It affects ecological properties of plant species and brings about instability of the ecosystem (Fangmeier et al. 2002; Walna 2007). Calculation of the correlation coefficient between acid-forming ions enables estimation of similarity of the time series of contaminants and the search for a common source. The highest correlation for acid-forming ions was in 2010, for both the open area and throughfall samples (Table 1). In 2011, only nitrates and nitrites showed high correlation coefficients with fluoride concentration. In the throughfall samples of 2010 and 2011, some correlation with phosphate was evident.

**Fig. 2:**
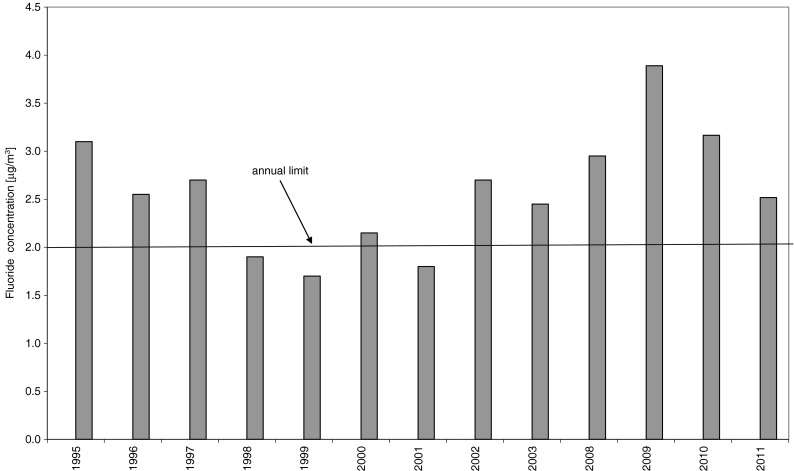
Comparison of annual average fluoride concentration in the air: years 1995–2011; *line* (2 μg/m^3^)—limit value for F concentration in the air

Average annual concentration of fluorides in air near the chemical plant emitting fluoride compounds (Ecological Report 2010/2011) has varied over recent years, often exceeding the allowable values (2 μg/m^3^; Fig. 2). The chemical plant is 10 km from Wielkopolski National Park where, owing to its special protection status, the permissible average annual fluoride concentration is 100 times lower (0.02 μg/m^3^). The daily average maximum fluoride concentration in air also approached the allowable limit (10 μg/m^3^), at 9.5 μg/m^3^ (2009) and 9.7 μg/m^3^ (2010). As stated earlier, however, the lowest concentrations that produce visible injury are around 0.3 μg/m^3^, if exposure time is sufficiently long (Cape et al. 2003).

**Fig. 3:**
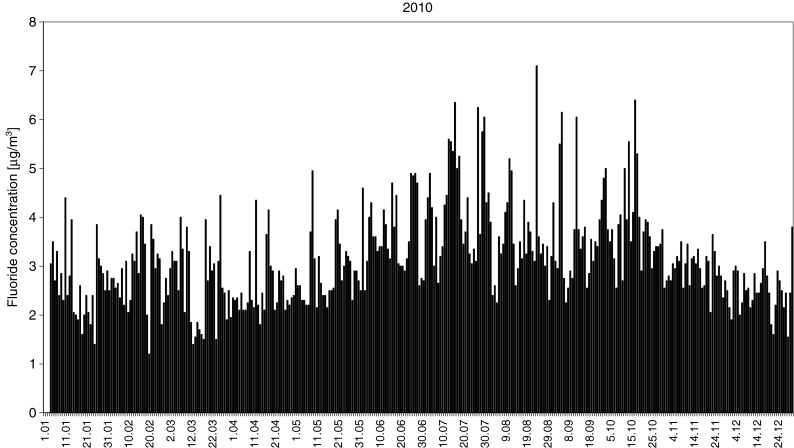
Fluoride concentration in the air in the vicinity of the study area in 2010

Annual variability of air pollution in 2010 is presented in Fig. 3. Twice, there were short breaks in measurements caused by an equipment failure (in January and March). Days of high atmospheric fluoride content and F^−^ concentrations in precipitation included 23 February 2010. Values on this day were 4.8 μg/m^3^ and 0.55 mg/l in the open area, respectively, and 0.98 mg/l in throughfall. Similarly, on 27, 28, and 29 July 2010, F content in the air significantly increased, exceeding 8 μg/m^3^. This was accompanied by high fluoride concentration in precipitation (0.34 mg/l). In 2011, high F content in the air was found on 10 and 11 May 2011 (4.35 μg/m^3^). This likely caused high F^−^ concentration in precipitation on 13 May 2011 (0.36 mg/l), as well as the 2.10 mg/l concentration in throughfall, which was the maximum in this year.

The atmospheric fluoride content near the chemical plant in Luboń was not sufficient to explain the substantial F^−^ concentrations in precipitation over the study area. Comparative studies of such concentrations in the city agglomeration 20 km north of the national park for 2003–2004 (Walna and Kurzyca 2007) showed the influence of other sources of fluoride emission. The increase in fluorides deposited over the last years, from 12.2 mg/m^2^ in 2003 to over 46 mg/m^2^ in 2011, indicates the need to look for the cause of such unfavorable changes.

The concentration of contaminants, including fluoride, is conditioned by atmospheric circulation, which transports the contaminants from their sources, and by the state of thermodynamic balance of inflowing air masses (Gasic et al. 2010; Scheringer 2009).

It was found that western and northern directions generally bring the most polluted precipitation (Table 2). In particular, the northeast direction brings the highest pollutant concentrations. Given that Poznań and the Luvena plant are at short distances northeast of the study area, one may assume these to be the major sources of pollution with advection from this direction.

**Table 2:** Chemical composition of rainwater according to the air-flow directions in accordance with Niedźwiedź’s (2011) classification (0—no prevailing direction); Wielkopolski National Park; years 2010–2011

	Cond	pH	F^−^	Na^+^	NH_4_^+^	K^+^	Mg^2+^	Ca^2+^	Cl^−^	NO_3_^−^	SO_4_^2−^	PO_4_^3−^	NO_2_^−^
	μS/cm	–	mg/l										
N	26.1	4.95	0.08	0.32	1.03	0.35	0.11	0.92	0.62	2.90	2.69	0.12	0.01
NE	**39.3**	**5.06**	**0.14**	**0.60**	**1.91**	**0.95**	**0.33**	**1.33**	**2.53**	**4.52**	4.34	0.04	**0.03**
E	21.4	4.71	0.06	0.23	0.71	0.40	0.07	0.61	0.53	2.08	2.23	0.08	<0.01
SE	30.1	4.44	0.08	0.34	0.83	0.17	0.10	0.76	0.75	3.03	3.07	0.16	<0.01
S	27.3	4.79	0.08	0.23	0.99	0.81	0.14	0.96	0.67	2.50	3.00	0.29	0.01
SW	37.1	4.70	0.12	0.44	1.37	**0.97**	0.16	1.04	1.26	**3.68**	**4.41**	**1.29**	0.01
W	36.6	4.98	0.12	0.41	1.41	0.88	0.18	**1.21**	**1.37**	3.51	**4.42**	**0.54**	0.01
NW	38.9	**5.10**	0.10	**0.51**	1.32	0.66	**0.18**	1.05	**1.37**	3.30	3.69	0.34	0.01
0	**39.1**	4.81	**0.13**	0.34	**1.57**	0.72	0.16	1.03	0.87	3.44	4.01	0.24	**0.03**

Values exceeding the third quartile have been marked in bold

Only certain pollutants, including fluoride, reach the highest concentrations with the presence of low or high pressure centers over Poland, without characterization by any prevailing direction (“0” according to Niedźwiedź’s classification in Table 2). Such synoptic situations are favorable for pollution from local emitters, including local domestic emission and from Poznań.

The 95th percentile criterion was applied to values of fluoride concentration in 134 rainwater samples collected in the open area during 2 years of research. It indicated 0.31 mg/l as a critical value. This allowed for selection of seven cases with extreme concentration of fluoride in rainwater. There were 5 days with fluoride concentrations higher than 0.31 mg/l in 2010 (23 February; 14, 23 and 29 July; 9 August) and only 2 days in 2011 (13 and 23 May; Fig. 1, Table 3).

**Table 3:** Episodes of extreme F concentration, dates of occurrence, and chemical composition

Precipitation event	Cond.	pH	F^−^	Na^+^	NH_4_^+^	K^+^	Mg^2+^	Ca^2+^	Cl^−^	NO_3_^−^	SO_4_^2−^	PO_4_^3−^	NO_2_^−^
	μS/cm	–	mg/l										
23 February 2010	261	3.21	0.55	1.61	6.52	0.96	0.75	2.96	5.35	19.8	34.5	0.12	0.02
14 July 2010	79.1	4.62	0.40	0.96	11.3	5.71	0.67	3.76	1.83	6.73	6.87	1.12	0.03
23 July 2010	27.0	4.77	0.32	0.17	2.06	0.35	0.13	0.97	0.31	4.05	1.95	<0.01	<0.01
29 July 2010	40.5	4.38	0.34	0.33	1.43	0.40	0.20	1.70	0.83	7.22	2.18	<0.01	<0.01
9 August 2010	53.5	6.58	0.51	0.54	3.10	0.61	0.28	4.20	0.95	7.54	3.65	0.81	0.05
13 May 2011	55.0	5.56	0.36	0.32	2.18	2.30	0.30	1.90	0.78	7.15	5.79	0.77	<0.01
23 May 2011	68.0	6.07	0.45	0.36	4.11	3.59	0.47	1.83	0.46	7.42	5.29	1.19	0.63

Following is a detailed description of synoptic conditions and airflow directions for the days with maximum concentrations of fluoride in precipitation.

### 23 February 2010

**Fig. 4:**
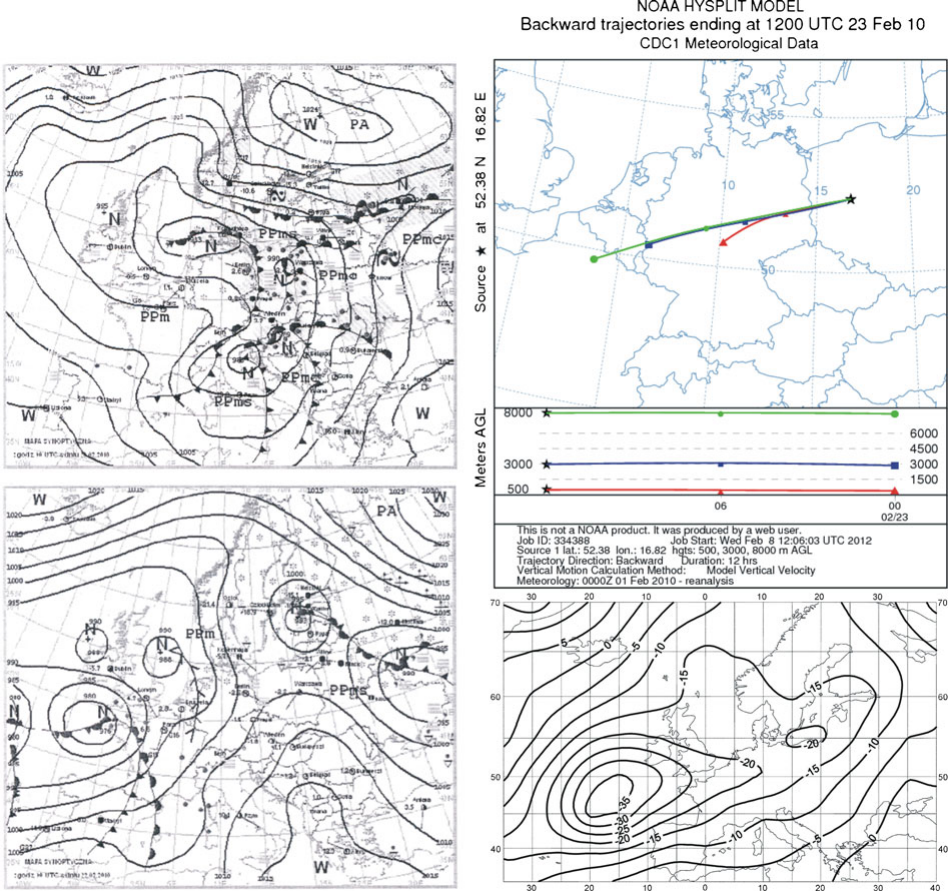
Synoptic situation for 19 February 2010 and 23 February 2010. *Left column*—sequence of two synoptic charts; *right column top*—24-h back trajectories at 500 m a.s.l. (*triangles*), 2,500 m a.s.l. (*squares*), and 5,000 m a.s.l. (*circles*); *right column top*—anomaly map of the sea level pressure

The highest fluoride concentration in precipitation during the 2 years was recorded on 23 February 2010. The synoptic situation on the day before this sample was influenced by a high pressure zone centered on the Mediterranean, which brought arctic sea air over Poland. It was cloudy in western Poland, with occasional snow and rainfall. Winds were weak and moderate, coming from the southwest sector (Fig. 4).

Twelve hours before the precipitation, air was flowing over the study area from the west. Results from the backward trajectory analysis at 500 m altitude reveal that the air entered from central Germany. The advection direction was similar at the other altitudes, and air at both traversed a similar distance from the German–French border through central Germany and over western Poland. The map of atmospheric pressure anomalies shows a southwest component of the advection (Fig. 4). The synoptic situation and back trajectories suggest that fluoride pollution in this case originated mainly from central Germany or western Poland. However, earlier synoptic maps (from 19 February; Fig. 4) show a low pressure center over Poland, along with a frontal system and clouds producing precipitation until the day of sample collection. Local circulation was controlled by that of the low pressure system and air advected over the study area initially from the east, then northeast, north, and finally the west. The localized low pressure generated precipitation, which probably originated in the vicinity of the study area. High concentrations of fluorides in precipitation could have come from the local production plants, where increased atmospheric air pollution by fluoride was observed at that time.

### 14 July 2010

**Fig. 5:**
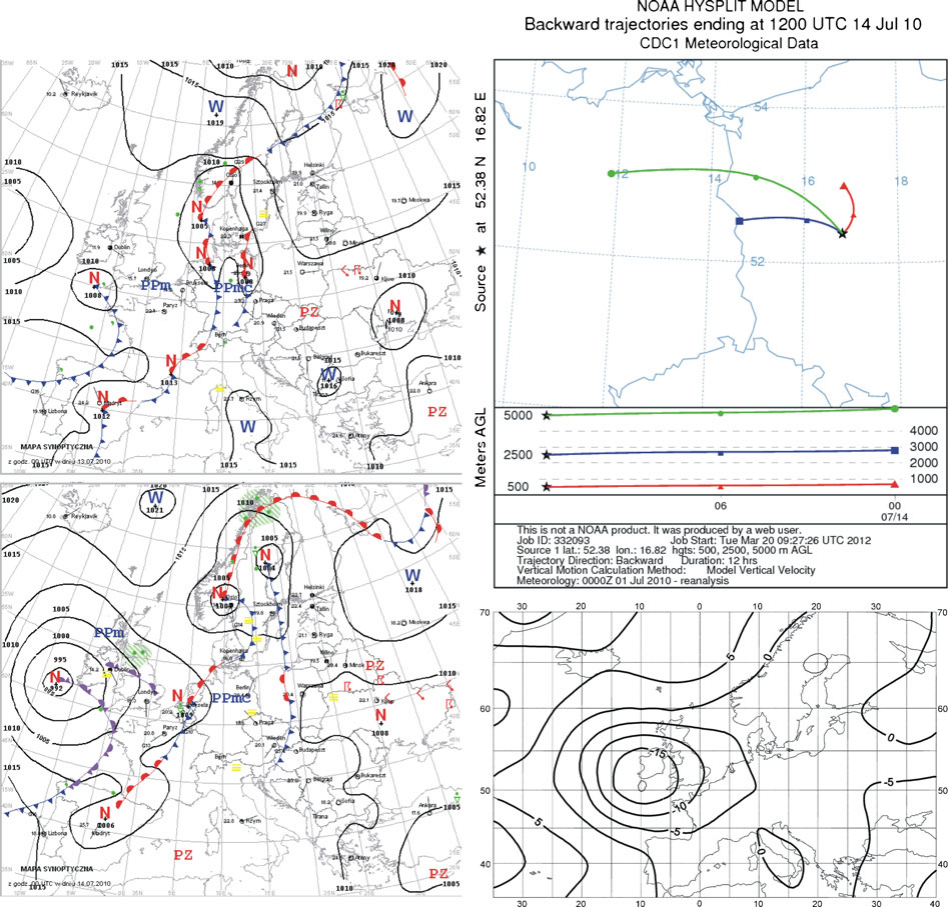
Synoptic situation for 14 July 2010. *Left column*—sequence of two synoptic charts; *right column top*—24-h back trajectories at 500 m a.s.l. (*triangles*), 2,500 m a.s.l. (*squares*), and 5,000 m a.s.l. (*circles*); *right column top*—anomaly map of the sea level pressure

On 13 July 2010, Poland was between low pressure zones from the Atlantic and southeast Europe. The west of the country was affected by a cool front, which was followed by slightly colder, humid arctic sea air. Cloud coverage increased to moderate and high. At many locations, there was rain and thunderstorms, with localized hail and heavy rain. The frontal zone separated hot tropical air over the eastern part of the country from the cold arctic sea air in the west. Winds were weak and changeable. The map of sea-level pressure anomalies shows a low pressure center over the British Isles and a weak low over the Black Sea (Fig. 5).

This synoptic situation generated air inflow from the northwest sector, as presented in the chart showing 12-h backward trajectories (Fig. 5). At each height in the chart, weak downward air motion is observed. In the mixing layer close to the surface, air motion was from the northwest-to-west sector (along the curved track), a short distance from the study area.

At 2,500 m, the air inflow direction was from the west, and air particles at that height reached the study area from western Poland. At the 5,000-m altitude, air entered from Germany. In this case, areas to the north or northeast of the sampling site may have been the pollution source, with fluoride compounds (0.4 mg/l concentration) transported over short distances. Part of the pollution could have originated from western Poland or Germany with the inflow at higher altitudes.

### 23 July 2010

**Fig. 6:**
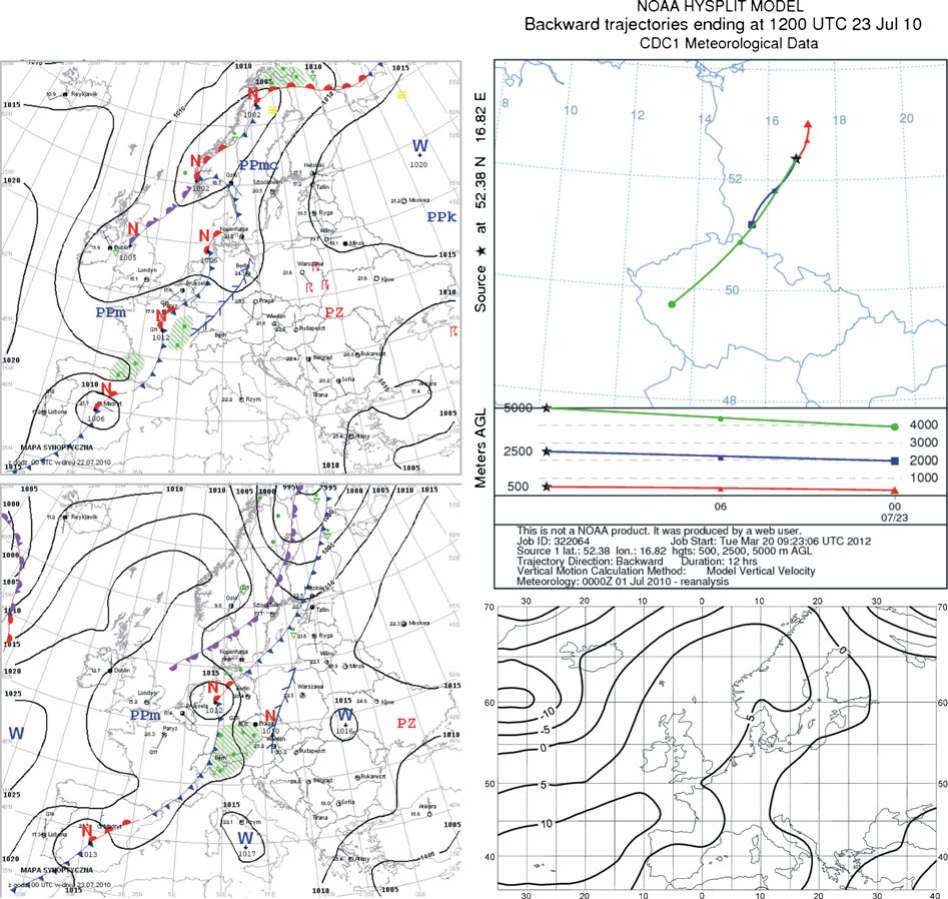
Synoptic situation for 23 July 2010. *Left column*—sequence of two synoptic charts; *right column top*—24-h back trajectories at 500 m a.s.l. (*triangles*), 2,500 m a.s.l. (*squares*), and 5,000 m a.s.l. (*circles*); *right column top*—anomaly map of the sea level pressure

Precipitation in July and August 2010 was characterized by a high content of fluoride compounds. The norm was exceeded in four cases (Fig. 1). The highest concentration from this period was on 23 July. On the day preceding the precipitation, western Poland was under the influence of a low pressure zone, with an active cold front moving from the west. The front was followed by cooler arctic sea air. Wind was moderate to strong, and gusty during storms. There was continuous rainfall in the west (Fig. 6).

Over 12 h in the mixed layer (500 m above ground level), air entered the study area from the north and northeast, from a short distance away. At the other altitudes, there was air inflow from the southwest, whereby air at 2,500 m moved from southwest Poland, and at 5,000 m from the Czech Republic (Fig. 6). Areas to the north or northeast of the sampling site might have been the source of pollution brought from short distances, while part of the pollution arriving with inflow at higher altitudes could have been transported from southwest Poland or the Czech Republic.

### 29 July 2010

**Fig. 7:**
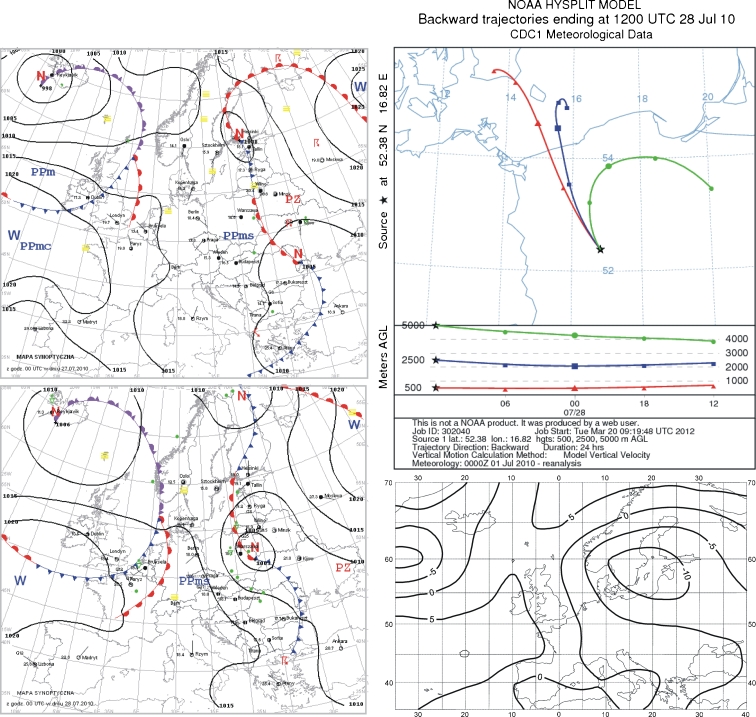
Synoptic situation for 29 July 2010. *Left column*—sequence of two synoptic charts; *right column top*—24-h back trajectories at 500 m a.s.l. (*triangles*), 2,500 m a.s.l. (*squares*), and 5,000 m a.s.l. (*circles*); *right column top*—anomaly map of the sea level pressure

Extremely high fluoride concentration in precipitation appeared again at the end of July 2010. On 28 July, Poland was under the influence of low pressure. There was total and high cloudiness, with sunny intervals in the west. There was occasional rainfall. Winds were weak to moderate, sometimes gusty, up to 17 m/s, from the north and west. During the night, Poland remained within the low pressure zone with its center over the southern Baltic Sea. On the day of sample collection, the country remained under the influence of this low pressure, within a zone of a transitory occluded front. From the west came cooler and humid arctic sea air. Winds were weak to moderate, from the west and southwest (Fig. 7).

During the 24 h before precipitation sampling, air at all heights entered the study area from the northern sector (Fig. 7). The source of these air masses was the southern Baltic Sea. The cause of pollution with fluoride was its emission from Polish areas to the north. Given atmospheric circulations at the center of low pressure, it is assumed that fluoride originated a short distance away, from the north or northeast. The probable emission source was the Luvena chemical plant, north of Wielkopolski National Park. This was confirmed by high fluoride concentration in the air on 29 July (7.7 μg/m^3^, Fig. 3; Report: Air quality in Luboń 2011).

### 9 August 2010

**Fig. 8:**
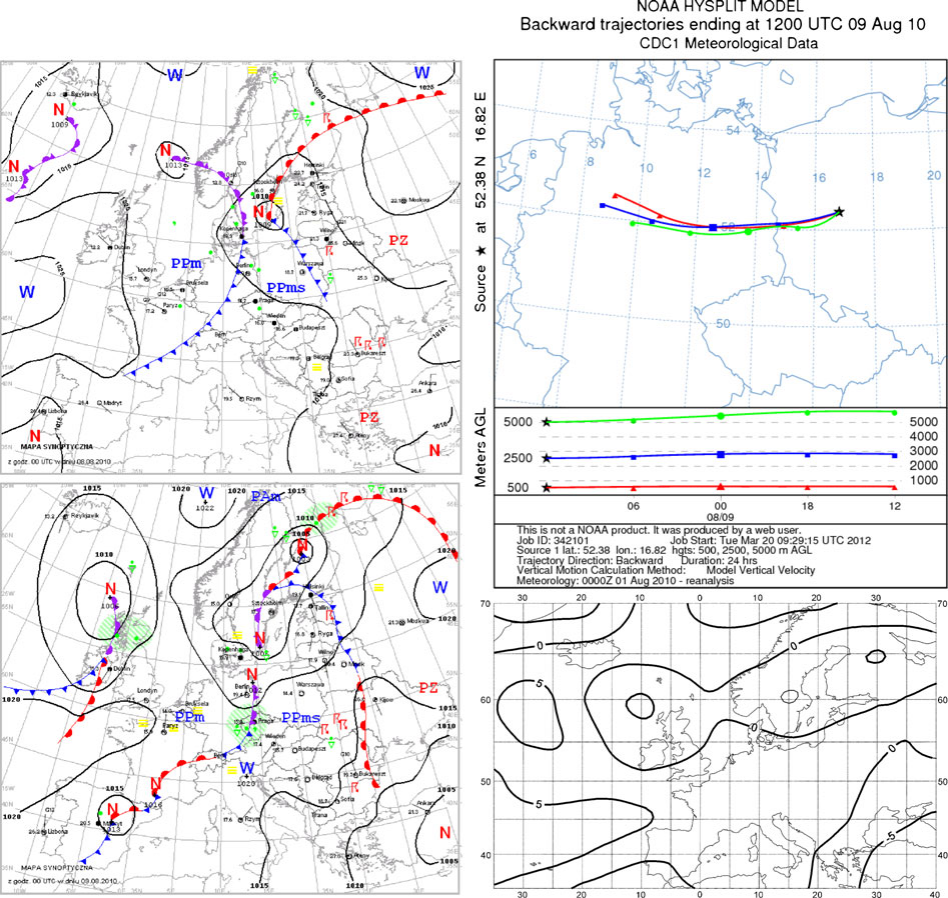
Synoptic situation for 09 August 2010. *Left column*—sequence of two synoptic charts; *right column top*—24-h back trajectories at 500 m a.s.l. (*triangles*), 2,500 m a.s.l. (*squares*), and 5,000 m a.s.l. (*circles*); *right column top*—anomaly map of the sea level pressure

On 8 August, Poland was affected by high pressure from Finland and northern Norway, with dry and cool continental air flowing from the east. Winds were weak to moderate, sometimes gusty. During the night, a warm atmospheric front moved over the center of the country. Warmer and humid air from the south entered Poland. On the day of sample collection, western Poland was under low pressure, in a frontal zone that produced periodical rainfall. Warmer and humid air advection continued from the south. It was cloudy, with sunny intervals locally (Fig. 8).

During the 12 h before the precipitation event, air entered the study area from the west (Fig. 8). During this time, air originating from northern Germany tracked the same distance at all altitudes. The cause of precipitation pollution with fluoride was its emission in Polish areas to the west or in northern Germany.

### 13 May 2011

**Fig. 9:**
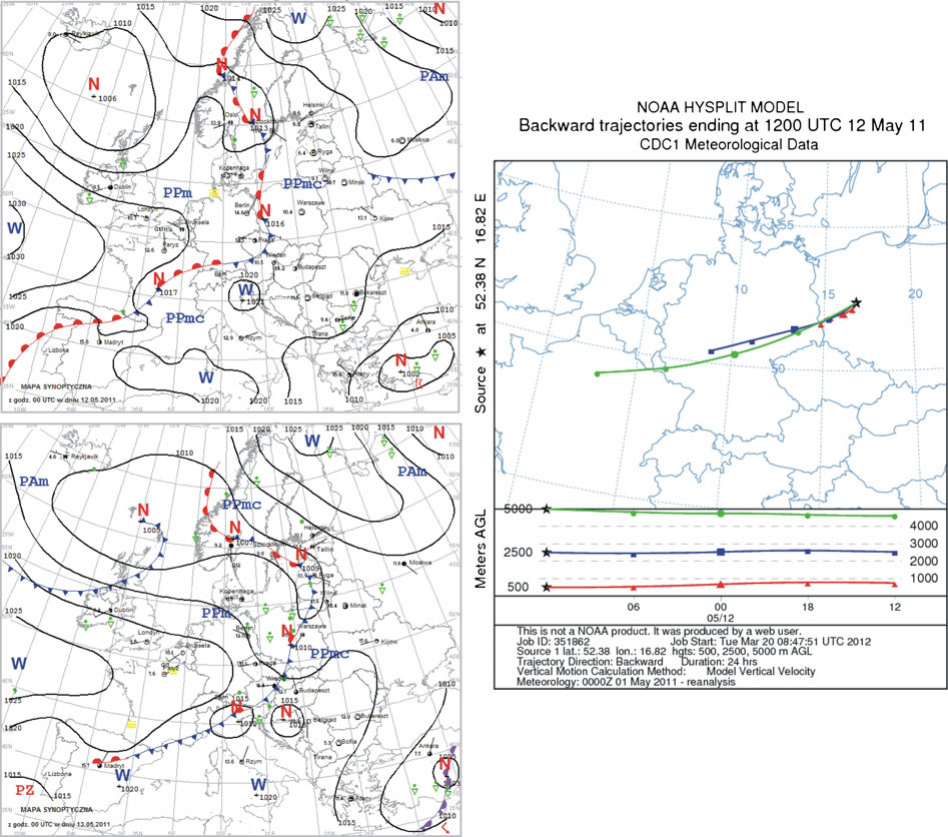
Synoptic situation for 13 May 2011. *Left column*—sequence of two synoptic charts; *right column*—24-h back trajectories at 500 m a.s.l. (*triangles*), 2,500 m a.s.l. (*squares*), and 5,000 m a.s.l. (*circles*)

On 12 May 2011, Poland was within reach of a low pressure zone with an associated front, moving from west to east. Winds were again weak to moderate, gusty during storms, coming out of the north. The front separated warm air in the eastern part of the country from cooler, arctic sea air in the west (Fig. 9). The next day, the frontal zone moved over the eastern part of the country, and high pressure from the Atlantic developed. Cool and humid arctic sea air masses came from the west. It was cloudy during the day, with sunny skies intruding from the west, but occasional rainfall (Fig. 9).

One day before sample collection, the trajectory of air motions at the three altitudes indicated a common direction of advection, from the southwest. The source area for air at altitude 500 m was southwest Poland. The air at 2,500 m came from Germany, and at 5,000 m from France (Fig. 9). However, similar to two previous cases (23 February 2010 and 29 July 2010), a low pressure center with atmospheric fronts developed over central Poland. This is the reason, despite the general circulation from the west, fluoride emitters within a relatively short distance from the study area should be investigated.

### 23 May 2011

**Fig. 10:**
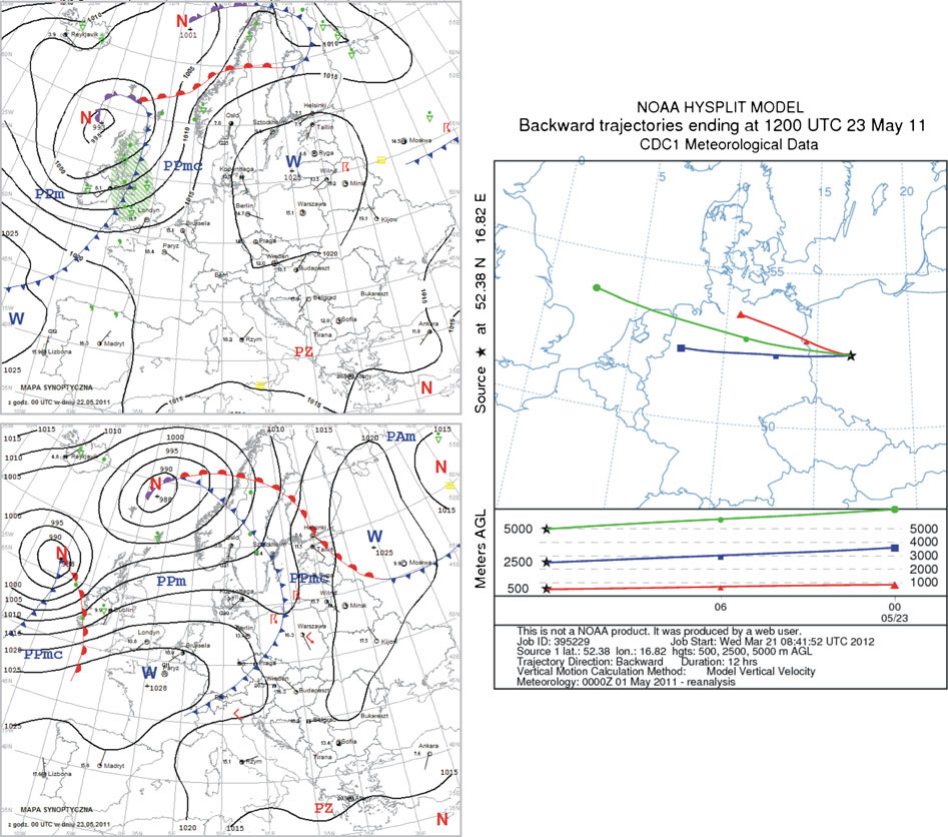
Synoptic situation for 23 May 2011. *Left column*—sequence of two synoptic charts; *right column*—24-h back trajectories at 500 m a.s.l. (*triangles*), 2,500 m a.s.l. (*squares*), and 5,000 m a.s.l. (*circles*)

Poland was on the edge of a high pressure zone from Latvia; in the afternoon, shallow troughs of low pressure began to enter the area. Very warm tropical air flowed in from the southwest. During the night, the low pressure troughs with a cool front moved from west to east, followed by cool, arctic sea air. Cloud cover was low to moderate, but at times more extensive with occasional rainfall and storms. Winds were weak to moderate, gusting to 16 m/s during storms, and directions were from the southeast, turning to the west. On the day of precipitation sampling, high pressure ridges developed over Poland. Cloud cover was low to moderate, increasing periodically. Winds were weak to moderate, from the northwest and west (Fig. 10).

In the 12 h before the precipitation, there were no significant differences in the direction of advection. In the mixing layer, the source area for the air was northern Germany. At 2,500 m above sea level, air moved in from western Germany, and at 5,000 m from the North Sea (Fig. 10). The fluoride pollution could have come from Belgium and Germany, as well as from western Poland.

## Conclusions

The 2-year study of fluoride levels in precipitation in Wielkopolski National Park revealed considerable concentrations, up to 0.55 mg/l in rainwater and 2.1 mg/l in throughfall. Analysis of daily data on F concentrations in the air indicated high values (maximum, 9.7 μg/m^3^), close to the allowable level. The possibly strong influence of fluorides on biotic and abiotic elements in the national park motivates the search for local and long-distance sources of pollution.

It was demonstrated in some studies that synoptic-scale atmospheric circulation patterns could not fully explain all aspects of pollutant transport, nor indicate source areas and forecast deposition locations (Dayan and Lamb 2005). Nevertheless, investigating only cases of extreme concentration of a selected pollutant may facilitate recognition of relationships between synoptic situations and severe rainwater pollution. We analyzed weather patterns preceding days with extreme fluoride concentrations in rainwater, in addition to airmass trajectories over the sampling site. This analysis produced the following conclusions:

- In all cases, weather conditions were controlled by the movement of weather fronts over western Poland, or by small cyclonic centers with fronts. The sampled rainwater came from frontal rainfalls, which were usually abundant and intense. Similar results were obtained from model analyses of the impact of mid-latitude weather events on atmospheric transport of chemical pollutants (Gasic et al. 2010). The potential for atmospheric transport increased during frontal events, as a result of efficient tropospheric mixing and high upper-tropospheric wind speeds. Frequent storms and strong wind speeds on the days of extreme fluoride pollution were manifestations of such dynamic atmospheric conditions.
- In most case studies, macroscale air advection over the sampling site originated from the western quadrant (NW, W, and SW), particularly in middle tropospheric layers (2,500–5,000 m a.s.l.). Such directions point to western Poland and Germany as possible pollution sources. In only one case (23 July 2010) was the Czech Republic suggested as the source. In the lower troposphere, air inflow was frequently from the north (three cases), suggesting short distance transport. In general, the western and northern directions bring the most polluted precipitation to the study area. Similar results were seen in analyses of global and synoptic scale weather patterns causing high sulphate pollution (Dayan and Lamb 2005, 2008; Salvador et al. 2010). It was shown that the greatest pollutant deposition occurs with intensified meridional flow over central Europe, indicating advection of cooler air from western Europe.
- The days with low pressure centers moving over central Poland are characteristic in terms of both direction and distance of advection in the lowest atmospheric layer. It is assumed that the source of fluoride pollution on these days was a short distance from the study area, and atmospheric circulations in the low pressure center transported fluoride from the north or northeast. Circulations on such a small scale could not be detected by the model used, with its 2.5° by 2.5° data resolution. In these cases, high pollution with fluoride may be linked to emission from the Luvena chemical plant, north of the national park.
